# Experimental Infection and Detection of Necrotizing Hepatopancreatitis Bacterium in the American Lobster *Homarus americanus*

**DOI:** 10.1100/2012/979381

**Published:** 2012-05-03

**Authors:** Luz A. Avila-Villa, Teresa Gollas-Galván, Marcel Martínez-Porchas, Fernando Mendoza-Cano, Jorge Hernández-López

**Affiliations:** ^1^Centro de Investigación en Alimentación y Desarrollo, A.C., P.O. Box 1735, 83304 Hermosillo, SON, Mexico; ^2^Aquaculture Department, Centro de Investigaciones Biológicas del Noroeste, Avenida Centenario No. 53, 83000 Hermosillo, SON, Mexico

## Abstract

Necrotizing hepatopancreatitis bacterium (NHPB) is an obligated intracellular bacteria causing severe hepatopancreatic damages and mass mortalities in penaeid shrimp. The worldwide distribution of penaeid shrimp as alien species threatens the life cycle of other crustacean species. The aim of the experiment was to evaluate the possibility of experimentally infecting the American lobster (*Homarus americanus*) with NHPB extracted from shrimp hepatopancreas. Homogenates from infected shrimp were fed by force to lobsters. Other group of lobsters was fed with homogenates of NHPB-free hepatopancreas. After the 15th day from initial inoculation, the presence of NHPB was detected by polymerase chain reaction in feces and hepatopancreas from lobsters inoculated with infected homogenates. Necrotized spots were observed in the surface of lobster hepatopancreas. In contrast, lobsters fed on NHPB-free homogenates resulted negative for NHPB. Evidence suggests the plasticity of NHPB which can infect crustacean from different species and inhabiting diverse latitudes. Considering the results, the American lobster could be a good candidate to maintain available NHPB *in vivo.*

## 1. Introduction

Necrotizing hepatopancreatitis bacterium (NHPB) is an intracellular organism causing hepatopancreatitis in penaeid shrimp [1, 2]. In early 1990s, morphological studies revealed the presence of different forms of NHPB, localized only in the cytoplasm and hepatopancreatic tubular epithelium of shrimp. Though morphologically they are markedly distinct, they could represent different life stages of a complex organism [1]. The bacterium is a member of the subclass *α*-Proteobacteria and has a severe pathogenic activity in the shrimp; for instance, recovery has not yet been observed in any shrimp after being infected with NHPB, and eventually the 100% have died [3, 4].

The bacterium is known to have severe physiological implications in crustaceans and have jeopardized the continuity of shrimp aquaculture in different countries [5]. Considering the above information and the fact that the white shrimp is one of the world's most widely cultured alien crustaceans [6], the introduction of shrimp pathogens into different environments is matter of concern.

To date, the presence of NHPB has been documented in tropical and subtropical ecosystems and a diversity of crustaceans can be hosts for the bacterium [1, 2, 5] such evidence suggests that NHPB could be highly tolerant to environmental challenges such as a wide range of temperature and, thus, thrive and be a threat in different environments. For instance, we have observed mortalities in American lobsters (*Homarus americanus*) that were accidentally fed NHPB-infected shrimp (unpublished data). However, the American lobster is a temperate-cold water crustacean and inhabits the Northeastern coast of the United States [7]. The aim of this experiment was to determine if the lobster could be experimentally infected with NHPB extracted from Pacific white shrimp (*Litopenaeus vannamei*).

## 2. Material and Methods

Healthy American lobsters *H. americanus *with an average weight of 1 kg were individually placed into a 40 L tanks containing filtered seawater. The experimental conditions were as follows: temperature 23°C, salinity 35‰, constant aeration, and daily water exchange of 80%. Lobsters were fed once a day with 5 g of fresh squid.

### 2.1. Inoculum Preparation

Infected shrimps (*Litopenaeus vannamei*) were experimentally inoculated with pure NHPB bacteria following the protocol described by Gracia-Valenzuela et al. [8]. After 3-4 weeks after inoculation, NHPBs were detected in hepatopancreas; thereafter, shrimps were sacrificed and dissected to extract the infested hepatopancreas.

Compositions of NHPB inoculums were based on hepatopancreas from infected shrimp. Hepatopancreas were extracted from shrimp and homogenized with glycerol (1 : 1 v/v) [9]. Before being supplied to lobsters, homogenates were confirmed positive to NHPB by polymerase chain reaction (PCR) tests in accordance with Nunan et al. [10]. A different homogenate was prepared using hepatopancreas NHPB-free and was considered as control. 

### 2.2. Forced-Feeding Infection of Lobsters

The treatment lobsters were infected with 1 mL of NHPB inoculum by forced feeding [11], and the inoculums was deposited into the oral cavity; the forced feeding was done by using an insulin syringe at 1st, 3rd, and 5th days from the beginning of the trial. Control lobsters were fed with NHPB-free inoculum by the same method. The feces of the treated and control lobsters were collected 15 days after infection; thereafter, the lobsters were sacrificed and the hepatopancreas were removed for further analyses.

### 2.3. Extraction of Genomic DNA and Detection of NHPB by PCR

Genomic DNA from lobster hepatopancreas was extracted by a commercial kit (Wizard SV Genomic, Promega, Madison, WI) following the manufacturer's specifications. Detection of NHPB-DNA was performed by PCR with primers NHPF2 5′-CGT TGG AGG TTC GTC CTT CAG T-3′ and NHPR2 5′-GCC ATG AGG ACC TGA CAT CAT C-3′ [10]. The reaction mixture contained PuRe Taq Ready-To-Go PCR beads (GE Healthcare, USA), 0.5 *μ*L of each primer (100 ng/*μ*L), 23 *μ*L of water, and 2 *μ*L of DNA template for reaction. The cycling parameters were performed under the following conditions: 95°C/2 min, 25 cycles of 95°C/30 s, 60°C/30 s, 72°C/30 s, and final extension at 60°C/1 min and 72°C/2 min.

PCR products were run in a 1.2% agarose gel, and the amplification products were visualized under UV light using the KODAK Imaging System program 4.0.

The PCR products were purified using a PCR purification kit QIAquick (QIAGEN, USA) following the manufacturer's specifications; thereafter, the purified samples were prepared and sent to a specialized laboratory (CISEI) to be sequenced. Nucleotide sequences were compared with the sequence U65509 GenBank in the algorithm Blast N of the National Center for Biotechnology Information Bethesda, MD (http://www.ncbi.nlm.nih.gov/BLAST/).

## 3. Results

After 15 days from the initial inoculation, NHPB was detected in both, feces and hepatopancreas, from lobsters fed by force with bacterial inoculums.

**Figure 1:**
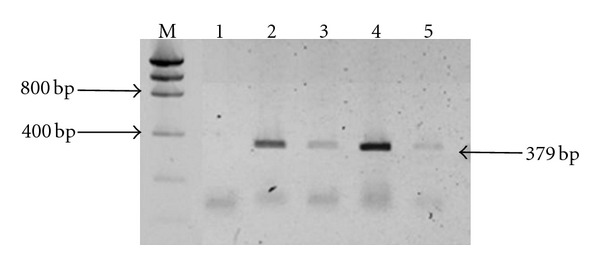
Agarose gel electrophoresis analysis of PCR amplification of extracted DNA from lobster feces. PCR was performed using Primers NHP/F2 and NHP/R2 [9]. Lane M indicates low-mass DNA marker, Lane 1 feces from control lobster, Lanes 2, 3, and 5 feces from treatment lobster, Lane 4 positive control NHPB of shrimp hepatopancreas.

**Figure 2:**
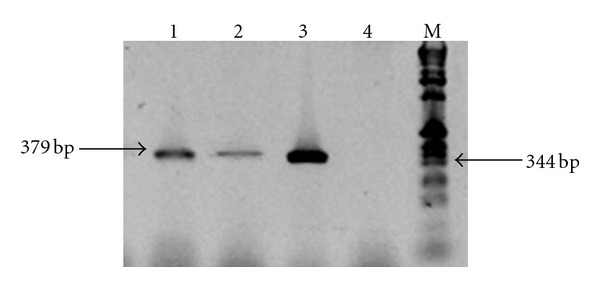
Agarose gel electrophoresis analysis of PCR amplification of extracted DNA from lobster hepatopancreas. PCR was performed using Primers NHP/F2 and NHP/R2 [9]. Lanes 1 and 2 indicate hepatopancreas from lobsters inoculated with NHPB, Lane 3 positive control NHPB of shrimp hepatopancreas, Lane 4 hepatopancreas from control lobster, Lane M 1 kb Ladder DNA marker.

**Figure 3:**
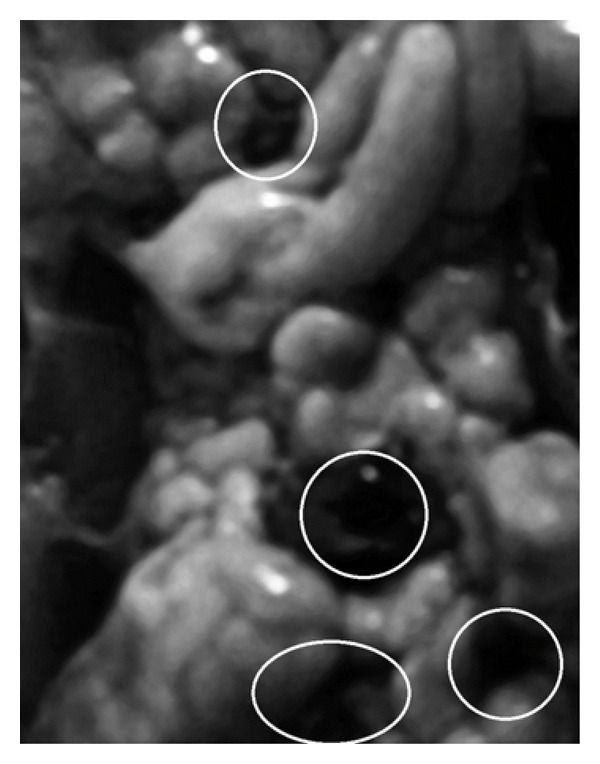
Hepatopancreas from lobster (*H. americanus*) experimentally infected with NHPB from penaeid shrimp (*L. vannamei*). White circles indicate blackish zones in which necrosis was observed.

The electrophoresis of the PCR products revealed 379 bp amplicons using NHPF2/R2 primers and genomic DNA (Figures 1 and 2). The results from sequencing the amplicons positive to NHPB (379 bp) from both, feces and hepatopancreas, were compared to the NHPB reference sequence of GenBank (U65509) and matched 100%. Additionally, blackish spots (2–5 mm) were observed along the hepatopancreas surface; these spots were necrotized areas (Figure 3).

Regarding lobsters inoculated with NHPB-free hepatopancreas, negative results were obtained for NHPB detection in both, feces and hepatopancreas. No spots were observed 15 days post infection on the hepatopancreas surface.

## 4. Discussion

Results strongly suggest that the American lobster can be a host for NHPB and that hepatopancreas malfunctions could occur during bacterial colonization.

The thermal conditions at which lobsters were maintained during the experimental period (23°C) are near the upper limit that American lobster has been observed (27.5°C) [11]. Considering that the thermal preference of the American lobster is around 18°C [7], the conditions of the experiment could have affected the resistance of the lobster and favored the propagation of NHPB. However, evidence has demonstrated that the thermal limits of the Pacific white shrimp and the American lobster can be overlapped [7, 12]. According to the Sea Life Base (http://www.sealifebase.org/), the distribution of the Pacific white shrimp has reached the East coast of the United States and apparently could be overlapped at some point with the distribution of the American lobster; however, a natural shrimp-lobster transmission has not been yet demonstrated.

The results of the experiment are also evidence of the NHPB plasticity, because it can colonize different species from different thermal ecosystems. Hence, the hypothetic plasticity of NHPB could be the reason why it is yet detected in countries from different latitudes.

Considering the above information, the worldwide distribution of white shrimp farms represents a risk for other crustacean species. White shrimp can be host not only for NHPB, but for a wide diversity of virus and bacteria [13]. Moreover, farms usually discharge effluents containing toxic metabolites and, in some cases, pathogenic microorganisms directly to the ecosystem [14, 15].

On the other hand, considering that *in vitro* methods have not yet been developed for NHPB culture and that the hepatopancreas size from lobster can be 40- to 50-fold greater than those of adult white shrimp, the American lobster may be an adequate candidate to maintain available NHPB *in vivo* [8].

In conclusion, the American lobster can be experimentally infected by NHPB, causing hepatopancreatic necrosis. This lobster can be a good experimental model to produce the bacteria *in vivo.* Results suggest the plasticity of NHPB to infect different crustacean species from different latitudes, but further studies are required to elucidate the infection process.
